# Circular Loop Ligation for Securing Endobronchial Valve in the Treatment of Bronchial Stump Fistula

**DOI:** 10.1155/carj/3080005

**Published:** 2026-08-26

**Authors:** Liping Wang, Jiagan Xu, Zhongliang He, Shunxin Xin, Congbin Peng, Weihua Xu

**Affiliations:** ^1^ Department of Pulmonary and Critical Care Medicine, Tongde Hospital of Zhejiang Province, Hangzhou, Zhejiang, China, zjtongde.com; ^2^ Cardiothoracic Surgery Department, Tongde Hospital of Zhejiang Province, Hangzhou, Zhejiang, China, zjtongde.com; ^3^ Anesthesiology Department, Tongde Hospital of Zhejiang Province, Hangzhou, Zhejiang, China, zjtongde.com

**Keywords:** bronchial stump fistula (BSF), bronchopleural fistula (BPF), bronchoscopy, circular loop ligation, endobronchial one-way valve (EBV)

## Abstract

**Background:**

Bronchial stump fistula (BSF), which is located on the bronchial stump following pulmonary resection, represents a distinct subtype of bronchopleural fistula (BPF). Although the endobronchial valve (EBV) has been used to treat BPF, few clinical studies involve BSF occluded by EBV because the abnormal bronchial stump makes EBV implantation challenging. The present research reports novel methods of using a circular loop ligation technique for securing EBV to achieve BSF occlusion and a new classification of BSF that is compatible with these novel methods.

**Methods:**

Based on the length of the bronchial stump, small visible BSF (SVBSF, less than 8 mm) is classified into the following types: SVBSF1 (< 3 mm), SVBSF2 (≥ 3 mm but shorter than a normal bronchus), and SVBSF3 (preserves a complete bronchial structure). SVBSF2 is further classified into SVBSF2a (the stump base remains partially intact) and SVBSF2b (complete destruction of the stump base). This retrospective research involved SVBSF2 patients who underwent EBV fixation with circular loop ligation. Detailed medical records were collected and analyzed to evaluate the efficacy of the novel procedure.

**Results:**

A total of 15 patients underwent EBV implantation and fixation. All patients achieved a resolution of air leakage. Postoperative assessment revealed no instances of EBV displacement among VBSF2a patients, with all cases exhibiting significant improvement.

**Conclusions:**

The circular loop ligation methods for securing the EBV enable effective BSF closure in SVBSF2, especially SVBSF2a cases. This technique enables some BSF patients with abnormal structures at bronchial stumps to successfully perform the EBV occlusion.

## 1. Introduction

Bronchopleural fistula (BPF) refers to an abnormal connection between the bronchus and the pleural cavity, with a mortality rate as high as 15%–71.2% [1]. Bronchial stump fistula (BSF) is a special type of BPF that occurs after pneumonectomy, lobectomy, segmentectomy, or subsegmentectomy, with the fistula located at the bronchial stump. Although some studies have explored the use of endobronchial valve (EBV) to occlude BPF [2–4], few clinical research involves BSF occluded by EBV because the abnormal bronchial structure of the stump—typically shorter than a normal bronchus—makes EBV occlusion of BSF challenging. The present manuscript reports the novel method of using a circular loop ligation technique to secure EBV for BSF occlusion and the new classification of BPF that is compatible with this novel method.

## 2. Methods

### 2.1. Classification of BPF

BPF is traditionally classified into central BPF and peripheral BPF based on location, with central BPF in postresection patients always being BSF. Recently, a special type of BSF called micro BSF (MBSF) was identified, which cannot be detected by direct bronchoscopic visualization and require guidewire exploration for diagnosis [5]. Based on this finding, BSF was further categorized into visible BSF (VBSF) and MBSF [5].

VBSF refers to the BSF that can be directly diagnosed under bronchoscopic visualization and is classified into two types according to its diameter [6]: larger VBSF (LVBSF, diameter ≥ 8 mm) and small VBSF (SVBSF, diameter < 8 mm). Because the outer diameter of EBV (version 5.5) is 8.5 mm, LVBSF cannot be occluded by EBV, while SVBSF may theoretically be occluded by it.

**FIGURE 1:**
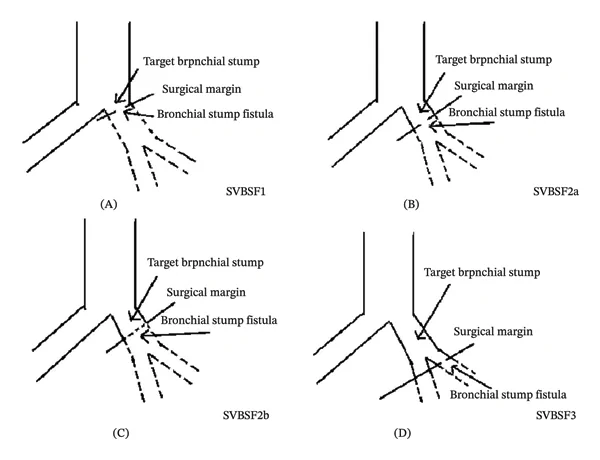
(A) SVBSF1, with the almost completely resected bronchus, leaving a stump shorter than 3 mm. (B) SVBSF2a, with the partially resected bronchus, leaving a stump shorter than a normal bronchus but longer than 3 mm, and the stump base remains partially intact, allowing EBV placement within the residual bronchus without risk of dislodgement into the ipsilateral pleural cavity. (C) SVBSF2b, with the partially resected bronchus, leaving a stump shorter than a normal bronchus but longer than 3 mm, and the stump base characterized by complete destruction, which predisposes the valve to migrate into the pleural space. (D) SVBSF3, with the complete bronchial structure, including intact subsegmental bifurcation. EBV placement is feasible for this type without requiring additional fixation method.

For SVBSF, we further classify it into the following types based on the length of the bronchial stump as shown in Figure 1.

 SVBSF1: The bronchus is completely or almost completely resected, leaving a stump shorter than 3 mm. BSF of this type cannot be occluded using an EBV due to insufficient residual bronchial length and lack of sufficient space to accommodate the EBV. When the EBV is attempted to be deployed in the target bronchial stump, it will be immediately displaced from the bronchial stump.
 SVBSF2: The bronchus is partially resected, with a stump shorter than a normal bronchus but longer than 3 mm. Although EBV placement is possible in BSF of this type, the shorter stump increases the risk of EBV migration, requiring additional fixation techniques to prevent inward displacement. SVBSF2 can be further classified into SVBSF2a and SVBSF2b based on the extent of damage at the bronchial stump base. In SVBSF2a, the stump base remains partially intact, allowing secure EBV placement within the residual bronchus without risk of dislodgement into the ipsilateral pleural cavity. In contrast, SVBSF2b is characterized by complete destruction of the stump base, which predisposes the valve to migrate into the pleural space. For effective EBV occlusion in SVBSF2b cases, dual fixation is essential: securing the valve on the bronchial side to prevent airway migration while also anchoring it on the pleural side to avoid thoracic cavity displacement.
 SVBSF3: The target bronchial stump retains a complete bronchial structure, including intact subsegmental bifurcation. EBV placement is feasible for this type without requiring additional fixation methods.

### 2.2. Patients Enrollment

The medical records of BSF patients at our hospital from September 2022 to November 2024 were retrospectively reviewed. The patients enrolled in the present study had to meet two conditions: (1) Their BSFs were identified as SVBSF2 and (2) these patients had received EBV placement and EBV fixation with circular coil ligation. Patients who had only undergone EBV placement but not EBV fixation were excluded.

The operations conducted in the present study were ethically approved by the Ethics Committee of our hospital, and informed consents were obtained.

### 2.3. Measurement of Bronchial Stump Length

The bronchoscope (Olympus, Japan, Model: BF‐1T260) was advanced into the airway until reaching the vicinity of the target bronchial stump. It was then further inserted into the bronchial stump until the tip reached its base. At this point, a mark was made on the external portion of the bronchoscope. Subsequently, the bronchoscope was gradually withdrawn until its tip aligned with the proximal end (starting point) of the target bronchial stump. A second mark was then made on the external portion of the bronchoscope. The distance between the two marks on the bronchoscope body corresponded to the length of the target bronchial stump.

### 2.4. EBV Placement and Fixation

Zephyr Endobronchial Valve (EBV, Pulmonx, USA, Model: ZEBV) was used to occlude the BSF in the present retrospective study.

A circular loop approximately 5 mm in diameter was created using surgical suture, with two 100‐cm‐long sutures attached at the 6 and 12o’clock positions of the loop. In this procedure, a nonabsorbable braided surgical suture (Ethicon 3–0; nominal diameter approximately 0.3 mm) was used for both the circular loop and the 100‐cm traction suture. These sutures were manufactured by Hangzhou Huawei Medical Supplies Co., Ltd. (Hangzhou, China). During the procedure, the loop was introduced into the bronchus through BSF from the thoracic side, while the distal ends of two attached sutures remained in the thoracic cavity to allow loop manipulation through traction.

If biopsy forceps could access the thoracic cavity through the BSF, they were used to grasp the loop from the thoracic side and position it either within the bronchus or guide it through the fistula and bronchus to exterior of endotracheal tube (depending on EBV ligation method), with distal ends of sutures remaining in thoracic cavity. When biopsy forceps could not access thoracic cavity via BSF, a guidewire was introduced through bronchoscope’s working channel into airway and advanced through BSF into thoracic cavity. The loop was then secured to guidewire with additional suture and guided to exterior of endotracheal tube, while loop’s attached sutures remained in thoracic cavity. Securing suture was then removed to free loop, which remains outside endotracheal tube. Alternatively, the loop may be pulled into bronchus by applying traction to thoracic‐side sutures (depending on the EBV ligation method).

**FIGURE 2:**
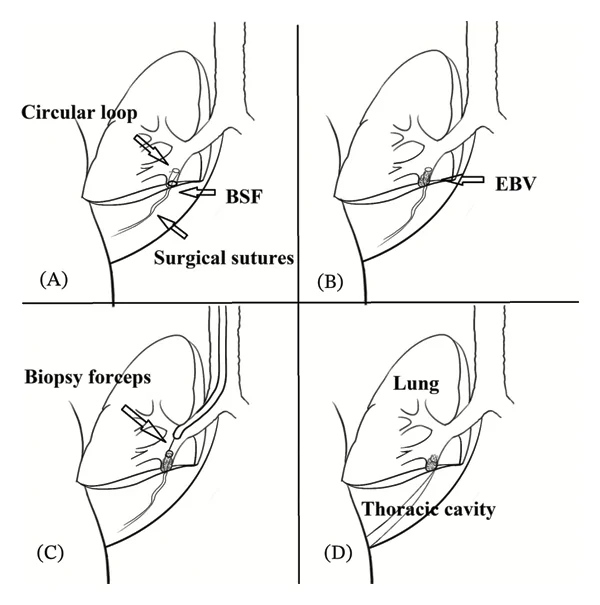
Loop‐assisted endobronchial valve fixation via a bronchoscopic approach. (A) The preassembled circular loop was advanced into the bronchus through the bronchial stump fistula (BSF) from the thoracic cavity. The distal ends of the two attached sutures were retained within the chest to enable subsequent loop manipulation by external traction. (B) Deployment of the endobronchial valve (EBV) was performed under direct bronchoscopic visualization. (C) Biopsy forceps were introduced through the working channel of the bronchoscope to grasp and position the preplaced circular loop around the neck of the deployed EBV. (D) The retaining sutures were progressively tensioned from the thoracic side, achieving secure EBV fixation through loop ligation. Finally, the suture ends were anchored to the chest wall.

**FIGURE 3:**
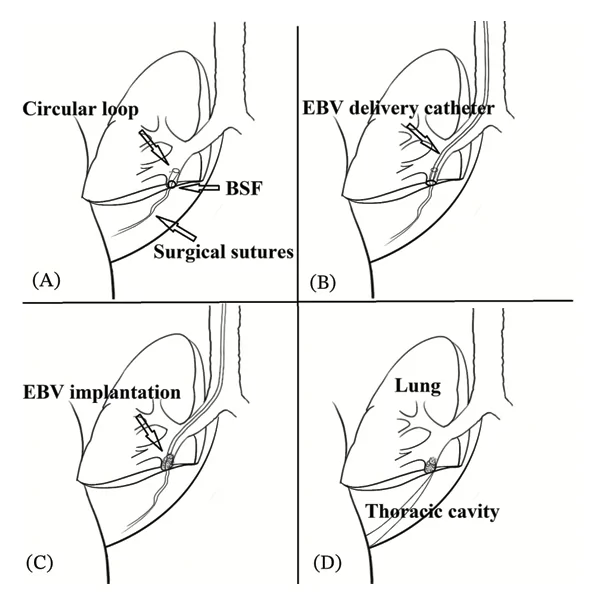
Synchronized loop ligation during endobronchial valve deployment. (A) The circular loop was introduced into the bronchial lumen through the BSF from the thoracic cavity. The distal ends of the attached sutures remained extracorporeally in the thoracic cavity to allow controlled traction. (B) The loaded EBV was advanced through the working channel of the bronchoscope toward the target bronchial stump. The delivery catheter traversed the prepositioned loop prior to reaching the intended deployment site. (C) Under real‐time bronchoscopic guidance, the EBV was precisely positioned to occlude the BPF. Simultaneous tensioning of the thoracic‐side sutures enabled immediate loop ligation around the EBV neck immediately upon device release. (D) Progressive suture tightening from the thoracic side ensured stable and secure fixation of the EBV via the encircling loop. Finally, the suture ends were sutured to the chest wall.

Three distinct methods were currently utilized for securing EBVs fixation using circular loop ligation as shown in the following:

 Method 1: As stated in the published paper [7], in brief, the EBV was loaded externally and deployed in the target bronchial stump under direct bronchoscopic visualization to occlude the BSF. Following withdrawal of the delivery catheter, biopsy forceps were introduced through the working channel of bronchoscope to grasp and position the preplaced circular loop around the EBV neck (Figure 2). The sutures were then progressively tightened from the thoracic side to achieve secure EBV fixation via loop ligation.
 Method 2: The externally loaded EBV was advanced through the bronchoscope’s working channel into the bronchus close to the target bronchial stump, where the delivery catheter first traversed the prepositioned loop before reaching the target bronchial stump. Under bronchoscopic guidance, the EBV was precisely positioned to occlude BSF while synchronized tightening of thoracic‐side sutures ensured immediate loop ligation around the EBV neck upon device release (Figure 3).
 Method 3: The distal end of the suture connected to the loop remained in thoracic cavity through BSF, with the loop placed outside the endotracheal tube. After loading the EBV externally, the EBV delivery catheter passed through the loop and advanced into the airway via endotracheal tube. By pulling suture from thoracic side, loop was synchronized with EBV delivery catheter to reach bronchial stump. Under bronchoscopic visualization, the EBV was deployed at bronchial stump to occlude BSF. Meanwhile, sutures were gradually tightened from the thoracic side, ensuring that loop ligature is secured around the EBV neck upon release.

Following successful EBV placement and circular loop ligation, the distal ends of the sutures connected to the loop were secured by suturing them to the parietal pleura. For SVBSF2b patients, due to the concurrent empyema caused by BSF, which led to infection of the tissues within the thoracic cavity, including around the fistula, EBV was not directly sutured at one side of the thoracic cavity’s fistula. Instead, a dural biological patch or pericardial biological patch was used to build a stabilizing base at the fistula site to prevent EBV from displacing into the thoracic cavity. The patch is sutured to relatively uninfected or less infected tissue on one side of the thoracic cavity with sutures to provide additional fixation support for the positioned EBV.

Postoperative patients with a chest tube were kept under continued observation. Follow‐up evaluations (outpatient or inpatient) were conducted for 6 months to assess clinical symptoms, the degree of air leakage from the chest tube, and repeat chest CT. If necessary, bronchoscopy was performed to evaluate the closure of BSF and the position of EBV. The main indicators for observation are the displacement of EBV and the relief of pulmonary air leakage.

### 2.5. Statistical Analysis

Data analysis was performed using SPSS Statistics software, Version 22.0. Nonnormally distributed data were presented as median and interquartile range [M (P25, P75)]. Categorical variables were expressed as frequencies and percentages (*n*, %).

## 3. Results

This study enrolled 15 patients with SVBSF2‐type fistulas who underwent EBV placement with circular loop ligation fixation, comprising 9 cases of SVBSF2a subtype and 6 cases of SVBSF2b subtype. The cohort comprised 9 males (60.0%) and 6 females (40.0%), with a mean age of 64 years (range: 29–74 years). The etiologies for lung resection were lung cancer (*n* = 10, 66.6%), pulmonary tuberculosis (*n* = 3, 20%), benign pulmonary nodule (*n* = 1, 6.7%), and bronchiectasis (*n* = 1, 6.7%). Baseline characteristics and outcomes of EBV placement in BSF patients are summarized in Tables 1 and 2. Nutritional status and pus cavity culture results of the patients are shown in Table 3.

**TABLE 1:** Basic information of patients in the present study.

Case	Sex	Age (year)	Etiology	Lung resection	Course of BSF (month)
1	M	74	Lung cancer	RLL	1.5
2	M	59	Bronchiectasis	LLL	108
3	F	65	Lung cancer	RLL	3
4	M	59	Tuberculosis	RLL	144
5	M	69	Lung cancer	RLL	1
6	F	61	Lung cancer	RLL	3
7	M	29	TB‐associated hemoptysis	RUL	12
8	F	64	Lung cancer	RUL	0.5
9	F	64	Lung cancer	RLL	1.5
10	M	61	Benign pulmonary nodule	RUL	26
11	F	47	TB‐associated hemoptysis	LUL	1
12	F	70	Lung cancer	RLL	1
13	M	67	Lung cancer	RLL	2
14	M	64	Lung cancer	LUL	0.5
15	M	72	Lung cancer	RUL	3

*Note:* M, male; F, female; EBV, endobronchial valve; TB, tuberculosis; LRL, left upper lobectomy.

Abbreviations: BSF, bronchial stump fistula; LLL, left lower lobectomy; RLL, right lower lobectomy; RUL, right upper lobectomy.

**TABLE 2:** Clinical data of BSFs, EBVs placement and fixation, and outcomes in the study.

Case	BSF size (mm)	BSF depth (mm)	BSF type	EBV fixation	EBV migration	Air leakage
1	2	5	SVBSF2a	Method 1	No	Cure
2	4	10	SVBSF2a	Method 1	No	Cure
3	4	4	SVBSF2a	Method 2	No	Cure
4	4	5	SVBSF2a	Method 1	No	Death
5	4	7	SVBSF2a	Method 2	No	Cure
6	3	5	SVBSF2a	Method 3	No	Cure
7	4	6	SVBSF2b	Method 3	No	Cure
8	4	7	SVBSF2a	Method 3	No	Cure
9	1	8	SVBSF2a	Method 3	No	Cure
10	4	5	SVBSF2b	Method 1	Yes	Recurrence
11	4	3	SVBSF2b	Method 1	No	Cure
12	5	7	SVBSF2b	Method 2	Yes	Recurrence
13	5	4	SVBSF2a	Method 2	No	Cure
14	6	4	SVBSF2b	Method 2	Yes	Recurrence
15	6	3	SVBSF2b	Method 3	No	Cure

Abbreviations: BSF, bronchial stump fistula; SVBSF, small visible bronchial stump fistula.

**TABLE 3:** Nutritional status and pus cavity culture results.

Case	NRS‐2002 score^∗^	Culture results of empyema	Serum albumin (g/L)
1	3	Negative	35.4
2	3	*Escherichia coli*	31.8
3	2	Negative	34.8
4	4	*Acinetobacter baumannii*	30.5
5	3	*Staphylococcus aureus*	33.7
6	2	Aspergillus	36.6
7	3	Negative	29.4
8	3	*Klebsiella pneumoniae*	35.5
9	2	*Pseudomonas aeruginosa*	33.1
10	2	Negative	32.8
11	3	Aspergillus	36.0
12	4	Negative	32.6
13	2	Negative	34.9
14	2	*Staphylococcus aureus*	35.8
15	4	*Pseudomonas aeruginosa*	31.7

^∗^NRS‐2002 score: Nutritional Risk Screening 2002 score. For the NRS‐2002 disease severity component, decortication of empyema was scored as 2 points.

Prolonged air leak and empyema had been present for substantial periods in all 15 patients before further thoracic surgical or interventional pulmonology procedures. Five patients had previously undergone unsuccessful bronchoscopic BSF closure attempts at other institutions before admission to our hospital, including two cases treated with conventional EBV placement, two cases with silicone stent implantation, and one case using an atrial septal defect occluder. These 15 patients demonstrated considerable variation in the time elapsed from BPF diagnosis to EBV placement with circular loop ligation fixation, averaging 3 months (range: 0.5–144 months). The average length of hospital stay was 18 days (ranging: 8–105 days).

BSFs were in the right upper lobe in 4 cases (26.7%), in the right lower lobe in 8 cases (53.3%), in the left upper lobe in 2 cases (13.3%), and in the left lower lobe in 1 case (6.7%). A total of 15 EBVs were implanted to occlude the BSFs, including 10 EBVs of size 4.0 mm and 5 EBVs of size 5.5 mm. In addition, using the previously reported method [8], three patients among the 15 patients were diagnosed with peripheral BPFs, which were located in the basal segment of the right lower lobe, the medial segment of the right middle lobe, and the lateral segment of the right middle lobe, respectively. All three peripheral BPFs were successfully sealed with Version 5.5 EBVs.

In our study cohort, EBV fixation using circular loop ligation was uniformly distributed across three technical approaches, with five patients undergoing each distinct method. All patients achieved resolution of air leakage following EBV implantation. Except for one SVBSF2a patient who died from severe aspiration pneumonia‐induced respiratory failure within two weeks postoperatively, all other patients completed over 6 months of follow‐up. None of the 8 SVBSF2a patients showed EBV migration, and all cases had persistent air leakage disappeared. With the treatment of empyema debridement, anti‐infection, and nutrition support, all patients had significant clinical improvement. Among the six SVBSF2b patients, three maintained a stable valve position, while the other three experienced EBV migration into the thoracic cavity, including one case where the EBV migrated together with a previously implanted atrial septal defect occluder.

Postoperatively, apart from the patient who died of aspiration pneumonia, another patient presented with a self‐limiting fever lasting two days. Of the 12 patients without EBV displacement, 2 developed local granulomas. Four patients reported increased coughing postsurgery, which responded well to oral cough suppressants.

## 4. Discussion

EBVs were primarily used for lung volume reduction surgery in patients with chronic obstructive pulmonary disease (COPD) [9]. The EBV consists of a silicone‐covered self‐expanding nickel–titanium alloy stent and an attached silicone one‐way valve flap. Its unidirectional opening effectively blocks airflow to specific lung regions, achieving occlusion [10, 11]. Subsequently, researchers have investigated novel applications of EBVs, including BPF closure [3, 12], management of persistent pneumothorax [13], and treatment of refractory hemoptysis [14].

The EBV is currently available in two models (EBV 4.0 and EBV 5.5) for adult patients, with treatment diameters of 4.0–7.0 and 5.5–8.5 mm, respectively. Obviously, for BSF with a diameter greater than 8 mm, the EBV cannot be used for occlusion. Therefore, in this study, we classified VBSF of 8 mm or larger as LVBSF and those smaller than 8 mm as SVBSF. This classification is consistent with those of other research [6, 15].

Additionally, EBV 4.0 and EBV 5.5 have the following dimensional specifications: the base‐to‐neck length measures 6.9 and 8.0 mm, respectively, and neck‐to‐tip length 4.7 and 5.2 mm, respectively, yielding total lengths of 11.6 and 13.2 mm, respectively. These well‐proportioned dimensions enable both valve models to be securely implanted in normal lobar, segmental and even subsegmental bronchi, thereby minimizing postprocedural displacement risks. Multiple clinical studies have demonstrated that the migration rate of EBVs placed in structurally normal bronchi remains consistently low, with an overall incidence of less than 5% [16, 17].

However, the bronchial stump structure in patients undergoing lobectomy, segmentectomy, or subsegmentectomy differs significantly from that of normal bronchi. This includes some BSFs almost without any bronchial stump, some BSFs with partial bronchial structure, and some BSFs with nearly complete bronchial structure. Based on these different characteristics, SVBSF may be classified into three types when considering EBV implantation to occlude BSF.

SVBSF Type 1 essentially lacks bronchial structure, presenting as a fistulous opening on a flat plane, making it unsuitable for EBV closure. For such BSF cases, coverage with the sidewall of a covered metallic stent or silicone stent proves more appropriate. For relatively larger‐diameter SVBSF Type 1 defects or LVBSFs, some researchers have employed atrial septal defect occluders or ventricular septal defect occluders for closure [18].

SVBSF Type 2 possesses a certain bronchial depth to potentially accommodate EBV placement for fistula closure. However, due to the inadequate length of the bronchial stump to fully contain the EBV, valve may easily displace. In the present retrospective study, two SVBSF2 patients had previously received EBV implantation but EBV consequently migrated into the airway. Using circular loop ligation to prevent bronchial‐side migration demonstrated excellent closure outcomes for SVBSF2a fistulas, with no EBV displacement observed during follow‐up of more than 6 months. In contrast, the SVBSF2b subtype lacks supporting structures at the stump base, predisposing it to thoracic cavity migration. For these cases, a dual‐anchoring approach combined bronchial‐side loop ligation with thoracic‐side fixation is necessary. Nevertheless, outcomes showed thoracic migration in 3 of 6 SVBSF2b cases, indicating a need for improved thoracic fixation techniques.

SVBSF Type 3 management is relatively straightforward as the preserved bronchial structure allows direct EBV implantation. Optional loop ligation can be added for enhanced stability.

After placing the EBV, we initially used biopsy forceps to grasp the loop wire, guiding it around the duckbill portion of the EBV and ligating it around the neck of the EBV. This method is relatively simple but often encounters some issues. For example, the loop wire sometimes adheres to bronchial secretions, causing the wire to stick together into a double‐stranded straight line, making it difficult to open. Repeated flushing with saline and using biopsy forceps to expand the loop are required to restore it to its circular shape. Second, when using biopsy forceps to grasp the loop wire for ligating the EBV, the loop often ends up ligating only a part of the EBV rather than forming a complete ring around the entire neck of the EBV, requiring repeated adjustments, which can be time‐consuming.

Subsequently, we improved the loop ligation method. The delivery catheter of the EBV was first threaded through the loop wire before being placed into the target bronchial stump. Meanwhile, the suture in the thoracic cavity was gently pulled to synchronize the loop ligation around the neck of the deployed EBV. This method reduced the difficulty of using biopsy forceps to grasp and ligate the EBV with the loop wire, but the issue of the loop adhering to secretions in the bronchus still persisted, requiring additional time to resolve.

Therefore, we further refined the loop ligation technique. The loop wire was first brought outside the airway, and the EBV delivery catheter was directly threaded through the loop wire outside the airway. Both were then advanced synchronously into the airway to reach the target bronchial stump. The EBV was then deployed, followed by loop ligation. This approach avoided the two problems of the first method but introduced its own challenges. For instance, when the loop wire and EBV delivery catheter enter the airway simultaneously, they must be coordinated precisely; otherwise, the catheter may slip out of the loop, requiring reoperation. Additionally, the loop wire and suture remain at the front end of the bronchoscope, potentially obstructing part of the visual field and necessitating careful positional adjustments.

In summary, for postresection patients with the complication of BSF, EBV implantation is a challenge because the bronchial stump is sometimes abnormally shorter than a natural bronchus. The application of loop ligation to secure EBV represents a novel approach that enables effective BSF closure in some BSF patients, especially those with SVBSF2a type. Based on the application of this innovative technique, a refined subclassification of BSF was further proposed for the first time. It must be stressed that, as this study is a single‐center retrospective analysis with a limited sample size, it has certain inherent limitations and needs further exploration in the future.

## 5. Conclusion

EBV implantation with fixation by circular loop ligation may be an effective method for BSF closure, especially for those SVBSF2a patients.

## Author Contributions

Liping Wang: writing the manuscript, investigation, and methodology.

Jiagan Xu: collecting the relevant clinical data and revising the manuscript.

Zhongliang He: designing the operation and methodology.

Shunxin Xin: investigation and methodology.

Congbin Peng: ensuring the accuracy or integrity of the operation and methodology.

Weihua Xu: designing the operation, final approval of the version to be published, and methodology.

## Funding

This research was supported by the Zhejiang Provincial Natural Science Foundation of China under grant number LGD21C040002 and funded by Key Traditional Chinese Medicine and Pharmacy Discipline Development Program of Zhejiang Province (2024‐XK‐05).

## Ethics Statement

This study has been registered in https://www.chictr.org.cn. The operations conducted in the present study were ethically approved by the Ethics Council Guide of our hospital and obtained consent. All study subjects signed informed consent prior to clinical interventional treatment, including the relevant medical data may be used in scientific research.

## Conflicts of Interest

The authors declare no conflicts of interest.

## Data Availability

The data that support the findings of this study are available from the corresponding author upon reasonable request.
